# Population-based comparative survival analysis of surgery with or without adjuvant radiotherapy and non-operative primary radiotherapy in patients with early-stage oral tongue squamous cell carcinoma

**DOI:** 10.1371/journal.pone.0259384

**Published:** 2021-11-11

**Authors:** Yu Jin Lim, Moonkyoo Kong

**Affiliations:** Department of Radiation Oncology, Kyung Hee University Medical Center, Kyung Hee University College of Medicine, Seoul, South Korea; University of Catania, ITALY

## Abstract

**Purpose:**

Although recent clinical guidelines do allow primary radiotherapy for selected patients with early-stage oral tongue cancer, there has been little knowledge on the treatment outcomes of non-operative radiotherapy using modern treatment techniques. This study evaluated recent prognostic differences between primary radiotherapy and surgical resection in T1‒2N0 oral tongue squamous cell carcinoma.

**Methods:**

Patients diagnosed with T1‒2N0 oral tongue squamous cell carcinoma were identified from the Surveillance, Epidemiology, and End Results database. After propensity score matching, the disease-specific survival of primary radiotherapy and surgery was compared.

**Results:**

From a total of 8,458 patients initially identified, we defined matched cohorts: cohort A, comparing surgery alone vs. primary radiotherapy (n = 230 vs. 230), and cohort B, comparing surgery plus adjuvant radiotherapy vs. primary radiotherapy (n = 230 vs. 230). The 7-year disease-specific survival rates were 77% vs. 35% (cohort A) and 65% vs. 35% (cohort B) (*P* < 0.001 for all comparisons). Primary radiotherapy was independently associated with worse disease-specific survival in both cohorts A (hazard ratio 4.06; 95% confidence interval 2.53‒6.52) and B (hazard ratio 2.81; 95% confidence interval 1.96‒4.04). Time-course hazard rate function plots showed a distinct short-term risk increment in disease-specific mortality in the primary radiotherapy group.

**Conclusion:**

In the contemporary treatment era, the use of radiotherapy as a definitive treatment resulted in an inferior prognosis in patients with T1‒2N0 oral tongue squamous cell carcinoma. The present population-based data suggest that primary radiotherapy cannot be used as an alternative to surgical management and it needs to be avoided as much as possible in early-stage tumors.

## Introduction

According to the recent cancer statistics report by the National Center for Health Statistics in the US, the mortality rate from oral tongue cancer has increased by approximately 2% per year, and 17,960 newly diagnosed cases are expected annually [1]. In contrast to locally advanced or metastatic tumors, patients with early-stage oral tongue cancer have a relatively good prognosis [2]. In general, the preferred management for stage I‒II tumors is surgical resection with or without cervical lymph node dissection or sentinel node biopsy [3].

In early-stage oral tongue cancer, optimal local control is essential for favorable long-term prognosis [4]. Most patients with T1‒2N0 disease are treated with surgical resection with or without postoperative radiotherapy (RT) according to pathologic adverse features, such as close or positive margins, perineural invasion, vascular invasion, and lymphatic invasion. However, recent clinical guideline does allow primary RT as a therapeutic option in early-stage oral cavity cancers [5]. The use of primary RT has been recommended as an alternative choice for selected patients who are not considered for surgery due to old age, medical comorbidities, or concerns about functional or cosmetic outcomes [6].

Nevertheless, no randomized data are available to compare surgical resection and primary RT in these node-negative early-stage oral cavity cancers. To date, only a few retrospective studies have reported data under contemporary RT planning methods [7–9]. Regarding technical advances over the last several decades, it has been expected that highly conformal RT would lead chances to expand the use of irradiation in clinics [10]. Therefore, it is necessary to evaluate the prognostic outcomes of primary RT in comparison with conventional surgical approaches in real-world practice.

We hypothesized that the long-term disease-specific survival of primary RT over recent years would be inferior to those of surgical resection in early-stage oral tongue squamous cell carcinoma. Based on a large-scale population-based database, this study evaluated the prognostic impact of primary RT using propensity score-matched comparative survival analyses. Since the postoperative risk related to treatment failure is not uniform among the patients treated with surgery, we defined two matched comparison sets: surgery alone vs. primary RT and surgery plus adjuvant RT vs. primary RT. This study provided an additional understanding of whether the use of RT is an alternative definitive management protocol in the contemporary treatment era.

## Materials and methods

### Study population

We obtained permission to access the Surveillance, Epidemiology and End Results (SEER) 18 database after signing the SEER Research Data Agreement [11] The SEER database currently covers approximately 35% of the United States population and comprises data from patients with cancer from 18 geographical registries [12, 13]. Personal information of registered patients was not included in the database and individual records were identified by assigning patient numbers. As this was a retrospective study of the data, the requirement for obtaining informed consent from patients was waived. Data extraction and manipulation procedures were in accordance with official guidelines [14].

**Fig 1:**
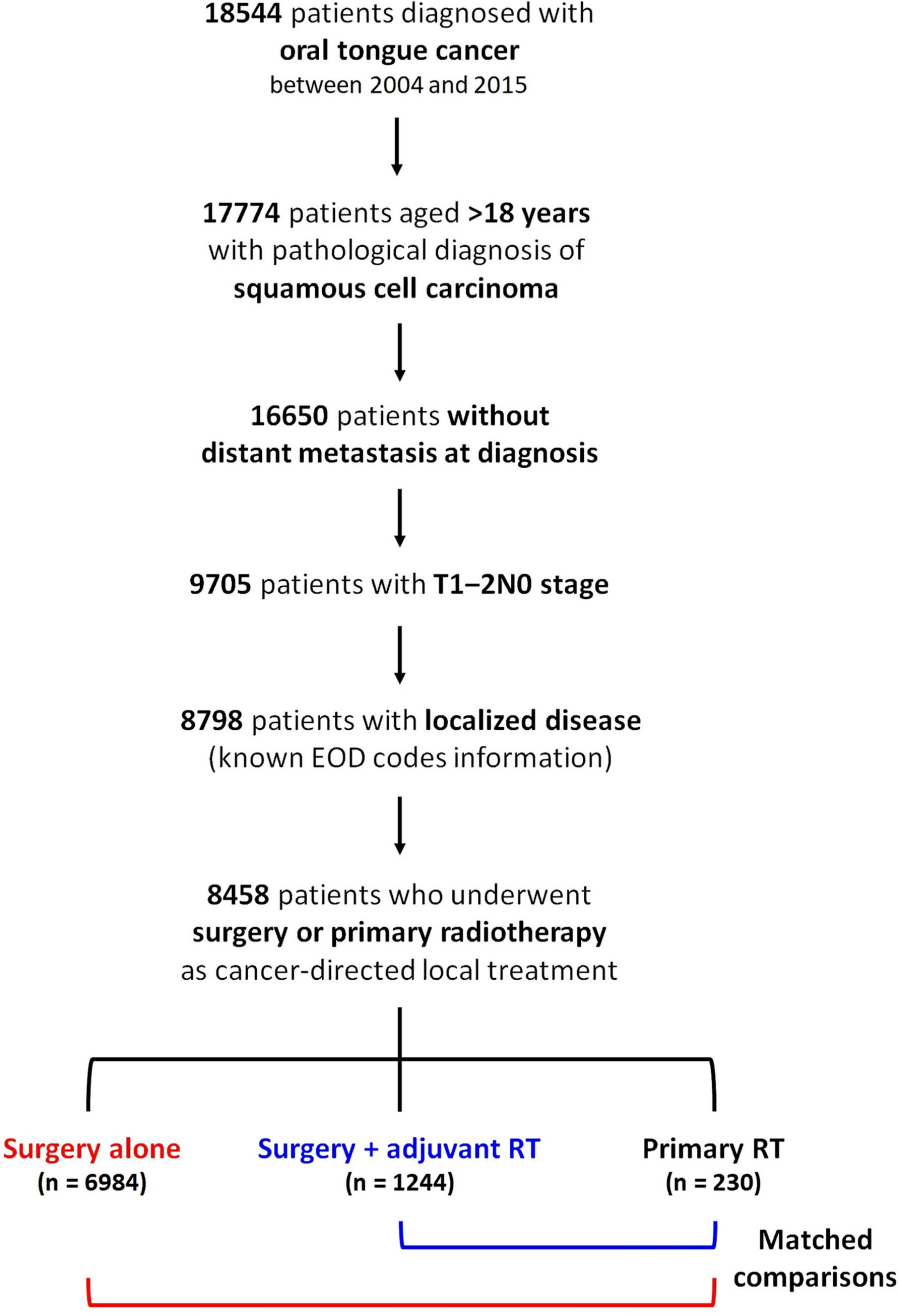
Patient selection process flowchart. RT: radiotherapy.

The raw data, including baseline characteristics and overall and cause-specific mortality information, were extracted using the case listing session of SEER*Stat software (version 8.3.6; National Institutes of Health, Bethesda, MD, USA) [15]. The “Site recode ICD-O-3/WHO 2008” variable was used to obtain cases with “tongue” as the primary tumor site. Squamous cell carcinoma was identified based on the third revision of the International Classification of Diseases for Oncology (ICD-O-3), with confirmed positive malignant histology codes. The eligibility criteria included patients with 1) pathologically diagnosed oral tongue squamous cell carcinoma; 2) year of diagnosis between 2004 and 2015; 3) age >18 years; 4) no distant metastasis; 5) early stage with T1‒2N0; 6) known information of extent of disease; and 7) cancer-directed local treatment with surgery (with or without adjuvant RT) or primary RT. Fig 1 shows the patient selection process flowchart.

### Propensity score matching

The cancer-directed local treatment was classified as surgery alone, surgery plus adjuvant RT, and primary RT. To estimate patient survival outcomes of primary RT in comparison with surgical resection, two propensity-matched comparison sets were defined: 1) surgery alone vs. primary RT (cohort A) and 2) surgery plus adjuvant RT vs. primary RT (cohort B). The propensity score is defined as the probability of being assigned to a treatment group [16]. Clinicopathological covariates available in the database were used for propensity-adjusted calculation and matched comparisons. Propensity scores were calculated with a non-parsimonious logistic regression model, and a one-to-one matching process was conducted based on the nearest-neighbor method without replacement. The final propensity-matched comparison set where the standardized difference (SD) values decreased for overall covariates was finally selected for survival analyses (acceptable if SD < 0.1) [17].

### Statistical analysis

The distribution of baseline variables was compared using the Pearson’s chi-square test and paired t-test or Mann‒Whitney U test for continuous and categorical variables, respectively. The primary outcome of interest was disease-specific survival (DSS). Overall survival (OS) and DSS were defined as the interval between the initial date of diagnosis and all-cause and tongue cancer-related death events, respectively. DSS was calculated for patients who were diagnosed with tongue cancer as the first malignancy. Survival outcomes were compared according to different local treatments based on Kaplan-Meier analysis with a log-rank test. After adjustment for potentially related covariates, multivariate analysis for independent prognostic factors was performed using the Cox proportional hazards model. Receiver operating characteristic (ROC) analysis was performed to evaluate the predictive ability of selected variables significantly associated with DSS. The area under the curve (AUC) and 95% confidence interval (CI) were calculated to estimate predictive accuracy. DeLong’s test was used to compare two ROC curves for different variables. The baseline hazard rate function plots for disease-specific mortality events were established using the R package “muhaz.” Two-sided *P*-values less than 0.05 were considered statistically significant. All statistical analyses were conducted using IBM SPSS Statistics 18 (IBM Corp., Armonk, NY, USA) and R version 4.0.2 (R Foundation for Statistical Computing, Vienna, Austria).

## Results

### Initial study population

**Fig 2:**
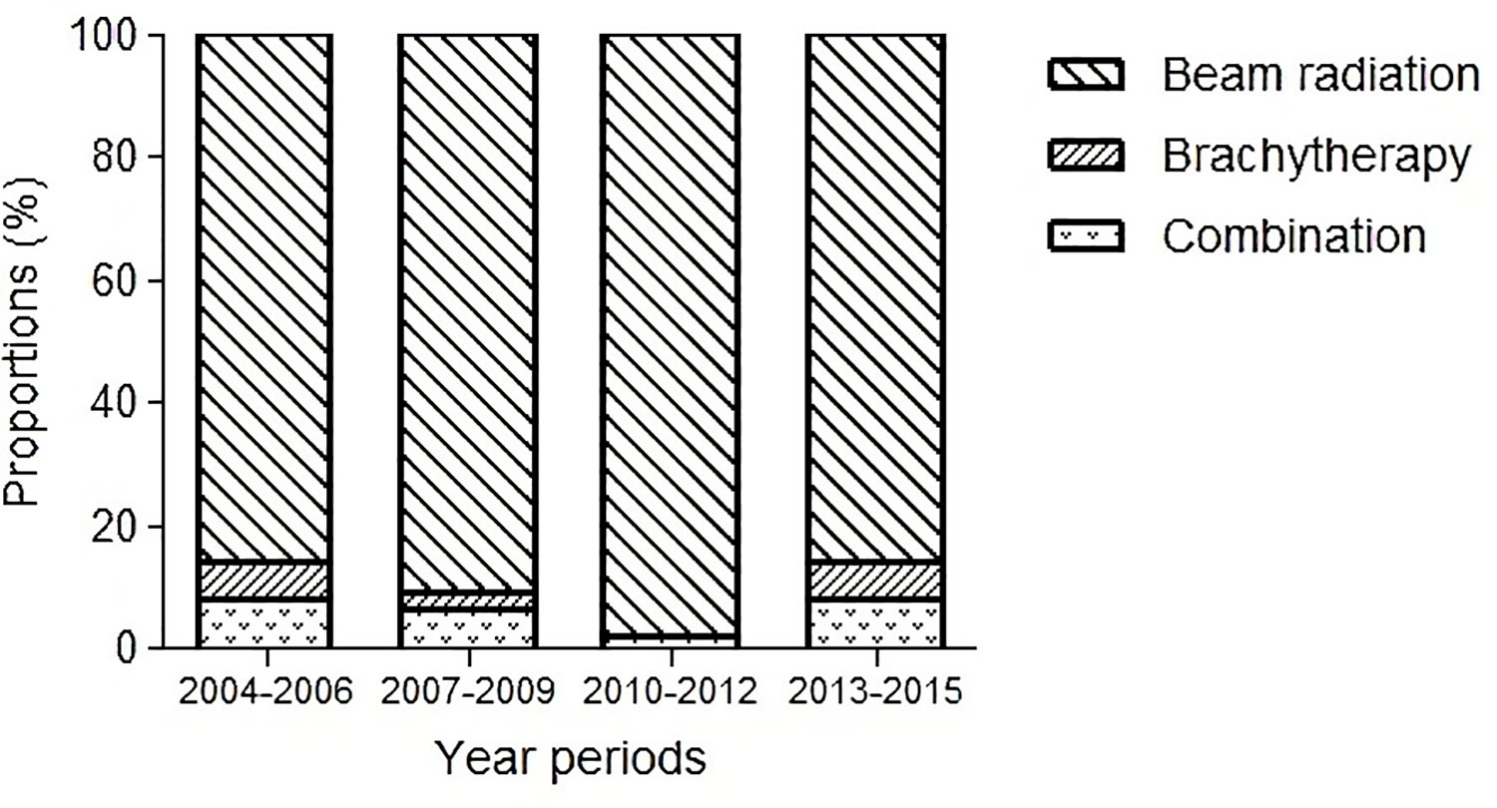
Changes in the proportions of irradiation methods (beam radiation, brachytherapy and combination of beam radiation with brachytherapy) over time periods in the primary RT group. RT: radiotherapy.

Initially, we identified 8,458 patients from the SEER database who met the eligibility criteria. S1 Table shows patient baseline characteristics. The median age was 63 years (range, 19‒105 years). Most of the patients were Caucasian (87%) with a male predominance (55%). Tumor grade II (48%) was the most common tumor type, followed by grade I (30%) and III‒IV (11%). The proportion of T1 tumors (75%) was greater than that of T2 tumors (25%). Tumor size information was available for 89.5% of the patients, with a median value of 1.5 cm (range, 0.1‒4.0). The primary tumor subsite was mostly specified at the border (25%) and anterior 2/3 (24%) of the tongue, followed by ventral (16%) and dorsal (5%) surfaces. In 42% of cases, the tumor was located on one side confined to the lamina propria or submucosa, while 23% of tumors extended into the musculature, intrinsic or not otherwise specified. In total, 6,984 (82%), 1,244 (15%), and 230 (3%) patients underwent surgery alone, surgery plus adjuvant RT, and primary RT, respectively. Approximately 91% of the patients in the primary RT group were treated with external beam radiotherapy (EBRT) alone, followed by EBRT combined with brachytherapy (6%) and brachytherapy alone (3%). Fig 2 illustrates the proportion of primary RT methods over time. The proportion of patients undergoing chemotherapy was 1% in the surgery alone group, 19% in the surgery plus adjuvant RT group, and 45% in the primary RT group.

### Propensity score-matched cohorts

Applying the propensity score-adjusted calculation, two matched cohorts were established: cohort A, comparing surgery alone vs. primary RT (n = 230 vs. 230), and cohort B, comparing surgery plus adjuvant RT vs. primary RT (n = 230 vs. 230) (S1 Fig). Table 1 shows the distribution of baseline characteristics after propensity score matching. Demographic and clinicopathological variables (age, sex, race, marital status, tumor grade, T stage, site of primary tumor, and extent of disease) available in the SEER database were analyzed. Regarding different clinical settings in terms of the use of chemotherapy with surgery or primary RT, the receipt of chemotherapy was not considered in the propensity score matching process; however, it was included as a covariate in the multivariate Cox regression model. Given the matching model, the SD values of all covariates were less than 0.1 in the matched cohorts A and B.

**Table 1:** Distribution of variables in the matched cohorts A (surgery alone vs. primary RT) and B (surgery plus adjuvant RT vs primary RT).

Characteristics	Matched cohort A [n (%)]		*Standardized* *difference*	Matched cohort B [n (%)]		*Standardized difference*
	Surgery (n = 230)	Primary RT (n = 230)		Surgery + adj RT (n = 230)	Primary RT (n = 230)	
Age (years)						
Mean ± SD	69.8 ± 12.9	69.3 ± 15.2	0.036	68.8 ± 13.1	69.3 ± 15.2	0.028
Sex						
Female	97 (42)	95 (41)	0.018	93 (40)	95 (41)	0.018
Male	133 (58)	135 (59)		137 (60)	135 (59)	
Race						
Caucasian	193 (84)	194 (84)	0.012	191 (83)	194 (84)	0.036
Others	37 (16)	36 (16)		39 (17)	36 (16)	
Marital status						
Married	104 (45)	100 (44)	0.014	100 (44)	100 (44)	0.007
Not married	109 (48)	115 (50)		116 (50)	115 (50)	
Unknown	17 (7)	15 (6)		14 (6)	15 (6)	
Grade						
I	43 (19)	43 (19)	0.053	29 (13)	43 (19)	0.062
II	107 (46)	104 (45)		123 (53)	104 (45)	
III‒IV	48 (21)	42 (18)		60 (26)	42 (18)	
Unknown	32 (14)	41 (18)		18 (8)	41 (18)	
T stage						
T1	74 (32)	70 (30)	0.038	76 (33)	70 (30)	0.057
T2	156 (68)	160 (70)		154 (67)	160 (70)	
Site of tumor						
Dorsal surface	15 (7)	18 (8)	0.087	14 (6)	18 (8)	0.015
Border	43 (19)	51 (22)		43 (19)	51 (22)	
Ventral surface	29 (13)	37 (16)		37 (16)	37 (16)	
Anterior 2/3	58 (25)	43 (19)		61 (26)	43 (19)	
Overlapping lesion	11 (5)	5 (2)		15 (7)	5 (2)	
Not otherwise specified	74 (32)	76 (33)		60 (26)	76 (33)	
Extent of disease						
One side confined to lamina propria or submucosa	29 (12)	39 (17)	0.035	28 (12)	39 (17)	0.010
Musculature, intrinsic or NOS	28 (12)	16 (7)		42 (18)	16 (7)	
Localized, NOS	61 (27)	67 (29)		50 (22)	67 (29)	
Crosses midline	55 (24)	50 (22)		47 (21)	50 (22)	
Invasion to adjacent structures^a^	57 (25)	58 (25)		63 (27)	58 (25)	

^a^Base of tongue, lower gingiva, floor of mouth, and sublingual gland were included.

RT: radiotherapy; adj RT: adjuvant RT; SD: standard deviation; NOS: not otherwise specified.

### Survival comparisons between the surgery and primary RT groups

**Fig 3:**
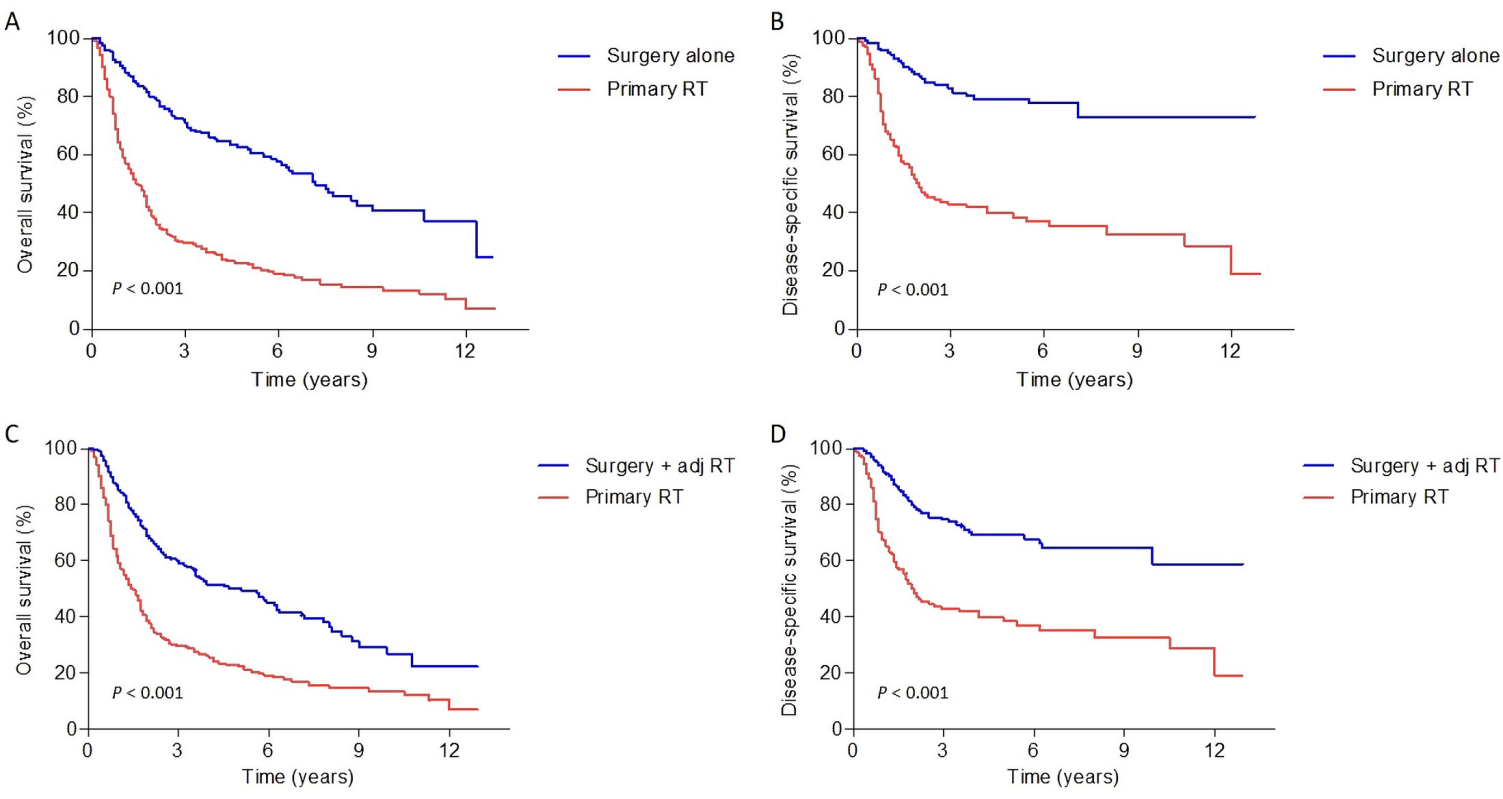
Overall and disease-specific survival curves when comparing surgery alone vs. primary RT (A, B) and surgery plus adjuvant RT vs. primary RT (C, D). RT: radiotherapy; adj RT: adjuvant radiotherapy.

In matched cohort A, the 5-year and 7-year OS rates in the surgery alone vs. primary RT were 62% vs. 23% and 53% vs. 17%, respectively, and the DSS rates were 78% vs. 40% and 77% vs. 35%, respectively (*P* < 0.001 for all) (Fig 3A and 3B). Comparing surgery plus adjuvant RT vs. primary RT in the matched cohort B, the 5-year and 7-year OS rates were 50% vs. 23% and 42% vs. 17%, respectively, and the DSS rates were 69% vs. 40% and 65% vs. 35%, respectively (*P* < 0.001 for all) (Fig 3C and 3D).

Among the entire study population that was initially identified, the elderly patients aged ≥70 years were additionally selected regarding the median age in the primary RT group. The DSS differences of the old-age patients were statistically significant. The 7-year DSS rates after surgery alone, surgery plus adjuvant RT, and primary RT were 81%, 57%, and 35%, respectively (*P* < 0.001 and *P* = 0.001 for comparisons of surgery alone vs. primary RT and surgery plus adjuvant RT vs. primary RT, respectively) (S2 Fig).

Table 2 shows the results of univariate and multivariate analyses for DSS. In univariate analysis of the matched cohort A, age > 70 years (vs. ≤ 70; *P* < 0.001), T2 stage (vs. T1; *P* = 0.009), tumor size > median 2.7cm (vs. ≤ 2.7cm; *P* = 0.001), primary RT (vs. surgery alone; *P* < 0.001), and receipt of chemotherapy (vs. no chemotherapy; *P* = 0.013) were associated with worse outcomes. After adjustment for the related covariates, age > 70 years (hazard ratio [HR] 2.12; 95% confidence interval [CI] 1.45‒3.11) and primary RT as the local treatment (HR 4.06; 95% CI 2.53‒6.52) showed an independently poor prognostic association. In matched cohort B, patients aged > 70 years (vs. ≤ 70 years; *P* = 0.011), tumor size > median 2.7cm (vs. ≤ 2.7cm; *P* = 0.075), and primary RT (vs. surgery plus adjuvant RT; *P* < 0.001) were associated with worse DSS and older age (HR 1.78; 95% CI 1.26‒2.53) and use of primary RT (HR 2.81; 95% CI 1.96‒4.04) remained significant in the multivariate analysis.

**Table 2:** Prognostic factors associated with disease-specific survival in matched cohorts A (surgery alone vs. primary RT) and B (surgery plus adjuvant RT vs. primary RT).

Variables	Cohort A				Cohort B			
	Univariate		Multivariate		Univariate		Multivariate	
	7-year rate (%)	*P*	HR [95% CI]	*P*	7-year rate (%)	*P*	HR [95% CI]	*P*
Age (years)								
≤ 70	62	< 0.001	Ref		52	0.011	Ref	
> 70	47		2.12 [1.45‒3.11]	< 0.001	49		1.78 [1.26‒2.53]	0.001
Sex								
Female	52	0.218			50	0.185		
Male	58				51			
Race								
Caucasian	56	0.743			51	0.389		
Other	56				47			
Marital status^†^								
Married	58	0.415			48	0.466		
Not married	52				51			
Tumor grade								
I	57	0.370			49	0.537		
II	52				55			
III‒IV	64				56			
T stage								
T1	65	0.009	Ref		58	0.123		
T2	51		1.48 [0.81‒2.71]	0.203	47			
Tumor size (cm)								
≤ 2.5	66	0.001	Ref		57	0.075	Ref	
> 2.5	47		1.39 [0.83‒2.31]	0.210	46		1.32 [0.93‒1.87]	0.117
Local therapy (cohort A)								
Surgery alone	77	< 0.001	Ref					
Primary RT	35		4.06 [2.53‒6.52]	< 0.001				
Local therapy (cohort B)								
Surgery plus adjuvant RT					65	< 0.001	Ref	
Primary RT					35		2.81 [1.96‒4.04]	< 0.001
Site of tumor								
Dorsal surface	48	0.291			42	0.693		
Border	48				55			
Ventral surface	69				61			
Anterior 2/3	55				54			
Overlapping lesion	76				65			
Extent of disease								
One side confined to lamina propria or submucosa	64	0.207			51	0.169		
Musculature, intrinsic or NOS	63				58			
Crosses midline	55				57			
Invasion to adjacent structures^a^	44				43			
Chemotherapy								
No	60	0.013	Ref		53	0.661		
Yes	41		0.74 [0.47‒1.16]	0.187	46			

^a^Base of tongue, lower gingiva, floor of mouth, and sublingual gland were included.

RT: radiotherapy; HR: hazard ratio; CI: confidence interval; Ref: reference; NOS: not otherwise specified.

### Comparison of risk factors with ROC curves

Based on the results of multivariate analyses, prognostic strength of age (> 70 vs. ≤ 70 years) and local treatment (primary RT vs. surgery alone or plus adjuvant RT) were compared using the ROC curves for disease-specific mortality (S3 Fig). In matched cohort A, the AUC values of age (> 70 vs. ≤ 70 years) and local treatment (primary RT vs. surgery alone) were 0.57 (95% CI 0.52‒0.63; *P* = 0.153) and 0.68 (95% CI 0.63‒0.73; *P* < 0.001), respectively. When the two ROC curves were compared, the difference in prognostic strength was statistically significant (*P* = 0.009). Comparing the curves in the matched cohort B, the difference was also significant (*P* = 0.011), with AUC values of 0.54 (95% CI 0.49‒0.59; *P* = 0.442) and 0.64 (95% CI 0.59‒0.69; *P* = 0.032) for age (> 70 vs. ≤ 70 years) and local treatment (primary RT vs. surgery plus adjuvant RT), respectively.

### Time-course risk changes in disease-specific mortality

**Fig 4:**
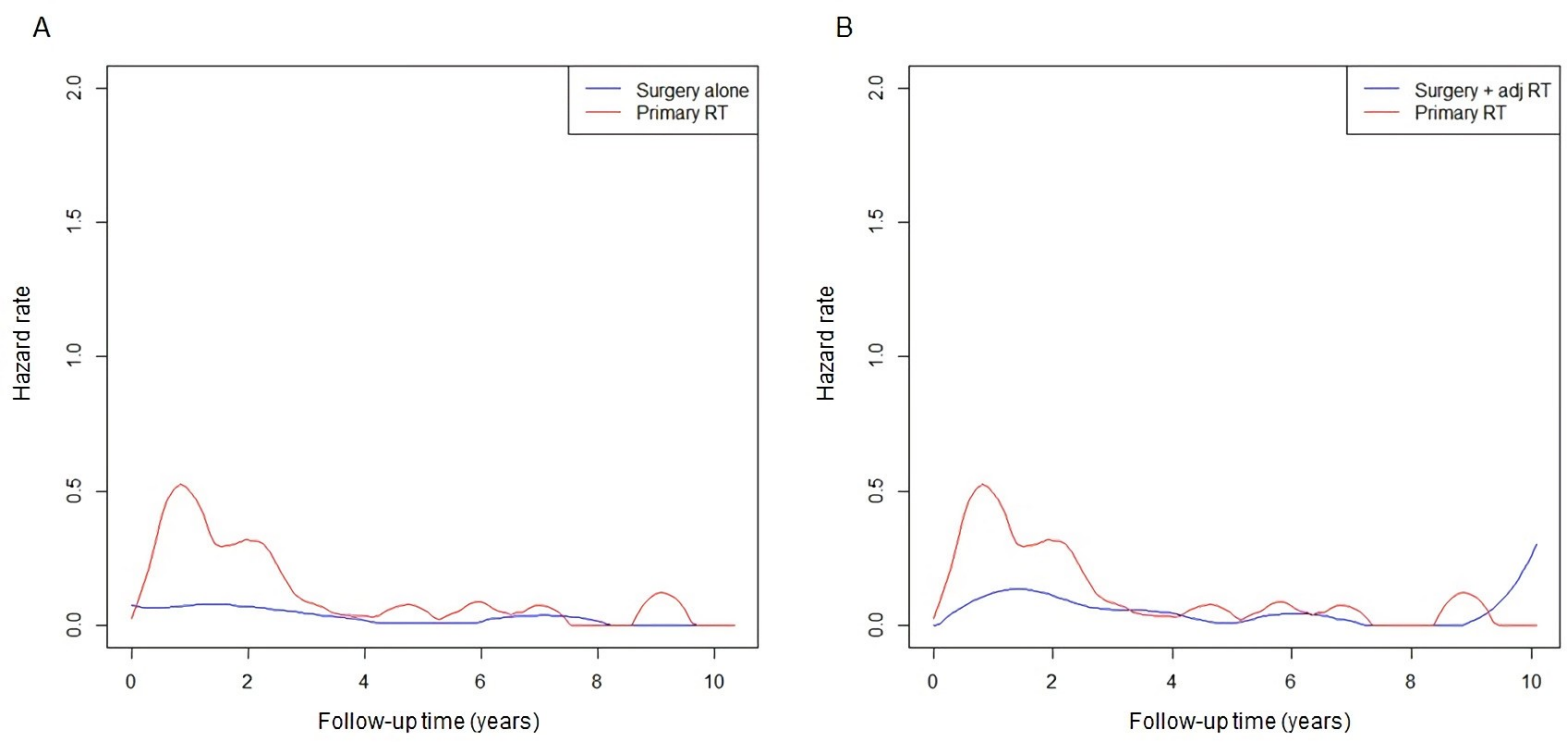
Hazard rate function plots of disease-specific mortality in the matched cohorts A (A) and B (B): surgery alone (*blue*) vs. primary RT (*red*) and surgery plus adjuvant RT (*blue*) vs. primary RT (*red*), respectively. RT: radiotherapy; adj RT: adjuvant RT.

The time-course hazard rate function plots for disease-specific mortality are shown in Fig 4. Patients who underwent primary RT showed a short-term risk increment within 3 years of RT, which was distinguishable from the other groups undergoing surgery. In the surgery alone group, there was a sustained lower risk level over the long-term follow-up period. In cases of surgery plus adjuvant RT or primary RT, a trend in increased disease-specific mortality occurring after approximately 8‒10 years of follow-up was observed.

## Discussion

This study used population-based long-term survival data to evaluate the prognostic impact of primary RT in early-stage T1‒2N0 oral tongue squamous cell carcinoma. In propensity-matched comparisons, patients who underwent primary RT had significantly worse DSS than those who underwent surgery alone or surgery plus adjuvant RT. The selection of primary RT showed a considerable association with poorer prognosis, even after adjustment for the effects of other covariates. In the time-course hazard rate function plots, the short-term risk surge of the primary RT group was distinguishable from that of the other groups undergoing surgery. Our matched comparison results provide additional insights into different prognoses according to the selection of definitive local treatment.

Patients with early-stage oral tongue cancer are mainly treated with partial glossectomy with or without cervical lymph node dissection or sentinel node biopsy. However, primary RT has been recommended in selected cases [18]. Old age, significant morbidity, and concerns about postoperative function or cosmetic outcomes are major factors to be considered when choosing non-operative RT, which can be inevitably encountered in real-world clinics [6]. The recent National Comprehensive Cancer Network (NCCN) guideline also states that definitive RT can be considered for T1‒2N0 oral tongue tumors, at the discretion of the physician [3]. Nevertheless, the limited number of patients undergoing primary RT has made it difficult to assess the potential role of this non-operative strategy in early-stage oral tongue squamous cell carcinoma [19]. To date, no randomized data are available directly relevant to this topic; some research groups have reported retrospective data. Owing to technical advances in modern RT over the last several decades, additional clinical data are required to update this topic.

Most historical survival outcomes following primary RT were based on small-sized institutional studies (Table 3) [20–29]. The prognosis of patients undergoing RT is considered comparable to that of surgical options on the basis of the reported 5-year OS rates, ranging from 60% to 80%, and the 5-year DSS rates, ranging from 75% to 90%. However, it is important to note that the majority of these studies involved patients undergoing brachytherapy alone or combined brachytherapy with EBRT, that is, not EBRT alone. On the basis of large-scale data, Rusthoven et al. first examined the results of the SEER analyses for stage I‒II oral tongue squamous cell carcinoma undergoing definitive RT between 1988 and 2004 [19]. The 5-year OS and DSS rates were reported as 60.9% and 83.5%, respectively, and different local treatment modalities among the patients were not considered. To our knowledge, few studies have addressed whether prognostic data mainly based on EBRT alone are comparable to surgical management in early-stage oral tongue squamous cell carcinoma.

**Table 3:** Institutional analyses of primary RT for early-stage oral tongue squamous cell carcinoma.

First author (year)	Institution (country)	No. of patients	Overall stage	Study period	RT modality and total dose (range or median dose)	5-year OS rates	5-year DSS rates
Leung (1993) [20]	Queen Elizabeth Hospital (Hong Kong)	117	T1‒2 N0‒3	1979 ‒1990	BT alone (58‒80 Gy): n = 51 BT (30‒70 Gy) + EBRT (10‒60 Gy): n = 14 EBRT alone (50‒80 Gy): n = 52	1) I: 81% 2) II: 67%	-
Lau (1996) [21]	British Columbia Cancer Agency (Canada)	27	T1‒3 N0	1989 ‒1993	BT alone (median 45.5 Gy)	66%	92%
Pernot (1996) [22]	Centre Alexis Vautrin (France)	565	T1‒3	1973 ‒1992	BT alone (66‒75 Gy)	1) T1: 70% 2) T2: 42%	-
Fujita (1999) [23]	Hiroshima University Hospital (Japan)	207^a^	T1‒2 N0	1980 ‒1993	BT alone (65‒70 Gy): n = 127 BT (50‒60 Gy) + EBRT (30 Gy): n = 80	1) T1: 83.4% 2) T2: 67.8%	1) T1: 90.1% 2) T2: 76.1%
Yamazaki (2007) [24]	Osaka University Hospital (Japan)	648	T1‒3 N0	1967 ‒1999	[According to HDR or LDR] BT alone (55‒78 Gy): n = 405 BT (39‒78 Gy) + EBRT (20‒44 Gy): n = 243	-	1) T1: 81‒86%^b^ 2) T2: 75‒81%^b^
Oota (2006) [25]	Tokyo Medical and Dental University Hospital (Japan)	277	II	1970 ‒1998	BT alone (70 Gy): n = 232 BT (60 Gy) + EBRT (30‒40 Gy): n = 45	-	89.5%
Guinot (2010) [26]	Fundacio´n Instituto Valenciano de Oncologı´a (Spain)	50	T1‒3	1999 ‒2007	BT alone (42‒49 Gy): n = 17 BT (12‒24.5 Gy) + EBRT (40‒70 Gy): n = 33	70%	-
Akiyama (2012) [27]	Osaka University Hospital (Japan)	51	T1‒2 N0	1996 ‒2004	BT alone (54 Gy or 60 Gy)	(3-year)^c^1) 54 Gy: 82% 2) 60 Gy: 88%	-
Matsumoto (2013) [28]	National Kyushu Medical Center and National Kyushu Cancer Center (Japan)	67	T1‒2 N0	1997 ‒2007	BT alone (40‒65 Gy): n = 33 BT (40‒65 Gy) + EBRT (7.5‒37.5 Gy): n = 34	88.7%	92.1%
Bansal (2016) [29]	Post Graduate Institute of Medical Education and Research (India)	92	T1‒2 N0	1999 ‒2014	BT alone (40‒52 Gy): n = 62 BT (18‒24 Gy) + EBRT (40 Gy): n = 30	73.2%	-

^a^Number of lesions.

^b^Survival outcomes according to HDR, Iridium-192, and Radium-226.

^c^3-year rates.

RT: radiotherapy; OS: overall survival; DSS: disease-specific survival; BT: brachytherapy; EBRT: external beam radiotherapy; HDR: high-dose rate; LDR: low-dose rate.

In this study of T1‒2N0 cases of oral tongue squamous cell carcinoma diagnosed between 2004 and 2015, only 9% of patients with primary RT were treated with radioisotopes. That is, definitive RT mostly comprising EBRT alone (91%) showed survival outcomes significantly inferior to those derived from previously reported small-sized studies listed in Table 3 [20–29]. The historical favorable outcomes determined in these studies were not based on the use of EBRT alone; therefore, it is not clear whether primary RT solely with the EBRT technique can be clinically recommended. The small proportion of cases of brachytherapy in our data would be related to the widespread use of advanced conformal EBRT techniques [30, 31]. Given the highly conformal irradiation methods used in clinics [32], such as intensity-modulated RT, volumetric modulated arc therapy, and Tomotherapy^®^, the preference for EBRT has increased over the past few decades. Orton et al. used the SEER database to evaluate trends in the use of primary RT modalities in cancer of the oral cavity and found that the rate of brachytherapy use continuously decreased by 0.58% per year (*P* < 0.001) [33]. Regarding the inferior outcome of primary RT from the population-based data, optimizing the use of RT modalities is necessary to enhance the therapeutic efficacy of definitive RT in patients for whom surgical resection is not medically recommended. Moreover, physicians need to recognize that the selection of primary RT may be an inferior treatment option with worse DSS than expected in early-stage oral tongue squamous cell carcinoma.

Our results derived from hazard rate function plots show that superior treatment efficacy within the initial years of follow-up is necessary to improve prognosis. With regard to short-term tumor control after definitive local treatment, we suggest that site-specific characteristics of oral tongue squamous cell carcinoma should be considered [19]. Since the oral tongue is movable within the oral cavity, early-stage oral tongue cancer lesions can be easily exposed through the mouth, which makes en bloc resection easier than in other head and neck subsites [34]. On the contrary, the accuracy of fixation or localization of the primary tumor site may be a concern in definitive RT [35]. The quality of radiation dose delivery with the EBRT technique may not be uniform across different institutions, but the variability in radiation dose delivery is not a significant issue with interstitial implant. Given the direct contact of radioisotopes with the tumor tissues, brachytherapy allows the maximum dose prescription for gross tumors with a rapid dose fall-off sparing adjacent organs at risk [36], suggesting that better local control is achievable with the use of radioisotopes [20]. Additional outcome analyses directly comparing the EBRT technique and brachytherapy are needed to confirm this hypothesis.

This study has several limitations. Propensity score matching was conducted to minimize the selection bias between different treatment groups; however, other potential bias from unknown covariates, such as performance status and general medical condition, still remains. Nonetheless, the registry includes more recent tumor, patient-related, and outcome records, and disease-specific mortality data were mainly analyzed as the primary outcome of interest to exclude death events irrelevant to tongue cancer diagnosis. Although this study alone cannot establish high-level evidence, we suggest that this hypothesis-generating study also provides useful insights with clinical implications. The predictive power of two prognostic factors for DSS was not sufficiently high in the ROC analyses, which might be attributable to methodological differences. The possible events of side effects and second primary tumors need to be assessed with respect to the therapeutic index of each treatment; however, the information was not available in the database. The lack of tumor recurrence data was an additional limitation.

In conclusion, this population-based study ascertained that primary RT, mainly based on EBRT alone, resulted in inferior survival outcomes compared to surgical management in patients with early-stage T1-2N0 oral tongue squamous cell carcinoma. In the multivariate analyses of DSS, the selection of primary RT was independently and strongly associated with a worse prognosis. Regarding the critical role of surgical resection as the initial definitive treatment for oral tongue squamous cell carcinoma, it is important to emphasize the inferior long-term outcomes under the contemporary use of RT. To enhance the therapeutic efficacy of RT as a local primary treatment for early-stage oral tongue cancer, the optimal radiation modalities to achieve a curative role should be established. Further prospective investigations are required to validate our results.

## Supporting information

S1 Fig Definition of matched cohorts A and B. RT: radiotherapy; adj RT: adjuvant RT.
(TIF)

S2 Fig Disease-specific survival curves of elderly patients aged ≥70 years when comparing surgery alone vs. primary RT (A) and surgery plus adjuvant RT vs. primary RT (B). RT: radiotherapy; adj RT: adjuvant radiotherapy.
(TIF)

S3 Fig Receiver operator characteristic curves of disease-specific mortality in the matched cohorts A (A) and B (B) comparing age (*red*) and local treatment (*blue*). ROC: receiver operator characteristic; Tx: treatment; RT: radiotherapy; adj RT: adjuvant RT.
(TIF)

S1 Table Clinicopathological characteristics of the initially identified study population with T1‒2N0 oral tongue squamous cell carcinoma (N = 8458). (DOCX)
