# Supplementary figures and images for: Population-based comparative survival analysis of surgery with or without adjuvant radiotherapy and non-operative primary radiotherapy in patients with early-stage oral tongue squamous cell carcinoma
Source: PLoS One. 2021 Nov 11;16(11):e0259384. doi: 10.1371/journal.pone.0259384 (PMC8584751; doi:10.1371/journal.pone.0259384)

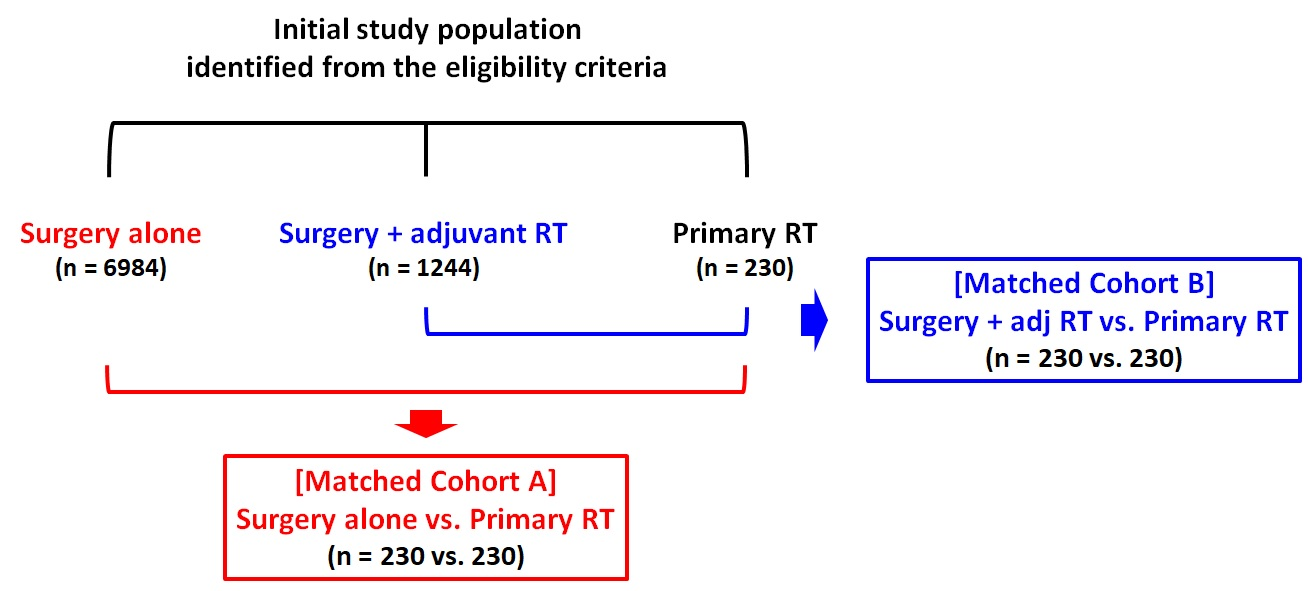

Supplement: S1 Fig — RT: radiotherapy; adj RT: adjuvant RT. (TIF) [file pone.0259384.s001.tif]

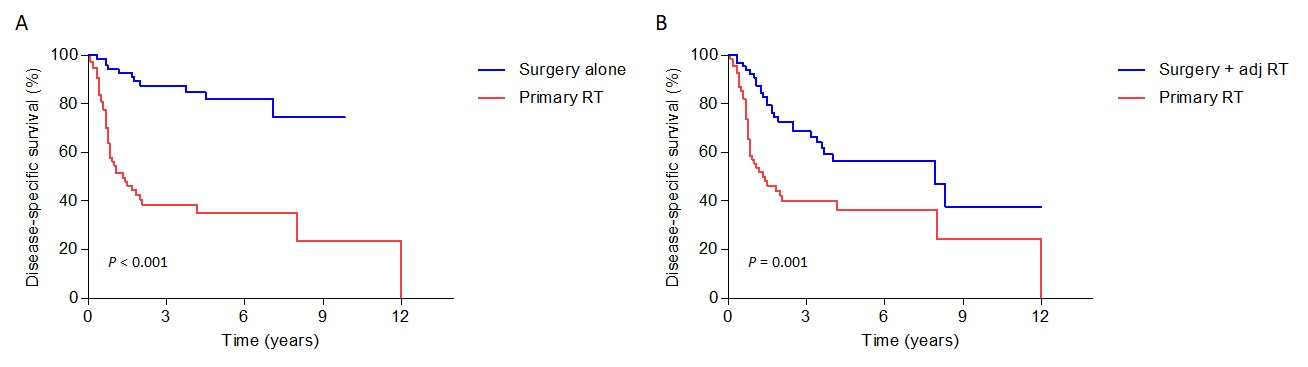

Supplement: S2 Fig — Disease-specific survival curves of elderly patients aged ≥70 years when comparing surgery alone vs. primary RT (A) and surgery plus adjuvant RT vs. primary RT (B). RT: radiotherapy; adj RT: adjuvant radiotherapy. (TIF) [file pone.0259384.s002.tif]

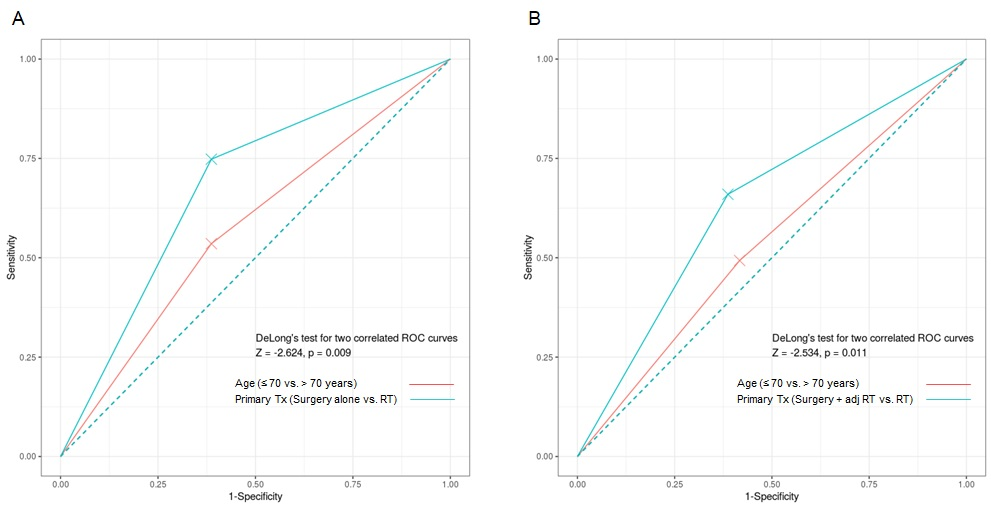

Supplement: S3 Fig — Receiver operator characteristic curves of disease-specific mortality in the matched cohorts A (A) and B (B) comparing age (red) and local treatment (blue). ROC: receiver operator characteristic; Tx: treatment; RT: radiotherapy; adj RT: adjuvant RT. (TIF) [file pone.0259384.s003.tif]
