# Supplementary material for: Population-based comparative survival analysis of surgery with or without adjuvant radiotherapy and non-operative primary radiotherapy in patients with early-stage oral tongue squamous cell carcinoma
Source: PLoS One. 2021 Nov 11;16(11):e0259384. doi: 10.1371/journal.pone.0259384 (PMC8584751; doi:10.1371/journal.pone.0259384)
Supplement: S1 Table — (DOCX) [file pone.0259384.s004.docx]

**S1 Table. Clinicopathological characteristics of the initially identified study population with T1‒2N0 oral tongue squamous cell carcinoma (N = 8458).**

| Characteristics | Number of patients (%) | | | | *P* |
| --- | --- | --- | --- | --- | --- |
|  | Total  (N = 8458) | Surgery alone  (N = 6984) | Surgery + adj RT  (N = 1244) | Primary RT  (N = 230) |  |
| Age (years) |  |  |  |  |  |
| Median (range) | 63 (19‒105) | 63 (19‒105) | 61 (19‒95) | 70 (29‒103) | < 0.001 |
| Gender |  |  |  |  |  |
| Female | 3825 (45) | 3185 (46) | 545 (44) | 95 (41) | 0.242 |
| Male | 4633 (55) | 3799 (54) | 699 (56) | 135 (59) |  |
| Race |  |  |  |  |  |
| Caucasian | 7376 (87) | 6114 (87) | 1068 (86) | 194 (84) | 0.007 |
| African American | 289 (3) | 653 (9) | 119 (10) | 21 (9) |  |
| Others | 793 (9) | 217 (3) | 57 (4) | 15 (7) |  |
| Marital status |  |  |  |  |  |
| Married | 4749 (56) | 3913 (56) | 736 (59) | 100 (43) | < 0.001 |
| Not married | 3083 (37) | 2505 (36) | 463 (37) | 115 (50) |  |
| Unknown | 626 (7) | 566 (8) | 45 (4) | 15 (7) |  |
| Tumor grade |  |  |  |  |  |
| I | 2540 (30) | 2286 (33) | 211 (17) | 43 (19) | < 0.001 |
| II | 4061 (48) | 3252 (47) | 705 (57) | 104 (45) |  |
| III‒IV | 949 (11) | 633 (9) | 274 (22) | 42 (18) |  |
| Unknown | 908 (11) | 813 (11) | 54 (4) | 41 (18) |  |
| T stage |  |  |  |  |  |
| T1 | 6309 (75) | 5648 (81) | 591 (47) | 70 (30) | < 0.001 |
| T2 | 2149 (25) | 1336 (19) | 653 (53) | 160 (70) |  |
| Tumor size (cm)^a^ |  |  |  |  |  |
| Median (range) | 1.5 (0.1‒4.0) | 1.3 (0.1‒4.0) | 2.2 (0.1‒4.0) | 3.0 (0.3‒4.0) | < 0.001 |
| Site of tumor |  |  |  |  |  |
| Dorsal surface | 455 (5) | 369 (5) | 68 (6) | 18 (8) | 0.020 |
| Border | 2074 (25) | 1714 (25) | 309 (25) | 51 (22) |  |
| Ventral surface | 1387 (16) | 1168 (17) | 182 (15) | 37 (16) |  |
| Anterior 2/3 | 2008 (24) | 1638 (23) | 327 (26) | 43 (19) |  |
| Overlapping lesion | 296 (4) | 238 (3) | 53 (4) | 5 (2) |  |
| Not otherwise specified | 2238 (26) | 1857 (27) | 305 (24) | 76 (33) |  |
| Extent of disease |  |  |  |  |  |
| One side confined to lamina propria or submucosa | 3578 (42) | 3211 (46) | 328 (26) | 39 (17) | < 0.001 |
| Musculature, intrinsic or NOS | 1968 (23) | 1546 (22) | 406 (33) | 16 (7) |  |
| Localized, NOS | 1764 (21) | 1438 (21) | 259 (21) | 67 (29) |  |
| Crosses midline | 762 (9) | 581 (8) | 131 (10) | 50 (22) |  |
| Invasion to adjacent structures^b^ | 386 (5) | 208 (3) | 120 (10) | 58 (25) |  |
| Chemotherapy |  |  |  |  |  |
| Yes | 391 (5) | 47 (1) | 240 (19) | 104 (45) | < 0.001 |
| None/unknown | 8067 (95) | 6937 (99) | 1004 (81) | 126 (55) |  |

^a^Cases with missing values were 10.5%.

^b^Base of tongue, gingiva lower, floor of mouth, and sublingual gland were included.

adj RT: adjuvant radiotherapy; RT: radiotherapy; NOS: not otherwise specified.
